# A Mediocrity Up-to-Date*Read before the North Texas Medical Association, December 8, 1908.

**Published:** 1909-03

**Authors:** Henry K. Leake

**Affiliations:** Dallas, Texas


For Texas Medical Journal.

### A Mediocrity Up-to-Date.*

BY HENRY K. LEAKE, A. M., M. D., DALLAS, TEXAS.

"If any, speak, for him have I offended."

On this occasion, I assume that your essayist shall speak of things that can be applied in the practice of medicine, or of medicine and surgery. Consequently, I am spared any attempt at a special discussion of social and political economics, or of commercial affairs which are held to bear a near kinship to the profession and which in many directions are being exploited, it is claimed, for the common benefit of the public and ourselves. Such relationships, if demonstrated, should be hedged about by safeguards that will define and regulate their wholesome limitations in order that no debasement of the profession shall ensue. The invitation to the profession to enter these fields should be accepted with reserve wisely imposed by the nature of the several parties to the contract.

Nor shall I discuss the relation that is believed to exist between religion, or, correctly speaking, ecclesiasticism and the medical profession. This association is known to be traditional, continuous and popular, but whether or not it should be perpetual I have no opinion to offer. If, as is contended by many, the union is indissoluble, be it so. These matters which, in the present at least,

*Read before the North Texas Medical Association, December 8, 1908.

have been examined and received as fixed principles may be considered to possess an up-to-date beneficence that it might be ruthless to disturb, notwithstanding reformation is possible always and finally may prove to be desirable. Let the experience of the past, the present and the future decide, as decide it will, at the behest of the evolution that inheres in and determines the fate of every manifestation of its ubiquity and power.

The modern physicians and surgeons are more intent upon the professional up-to-date views and practices and in this respect they occupy in history a unique and advanced position. From rapid inter-communication, post-graduate medical schools, numberless societies, journals and clinics they are made the daily recipients of an elaborate knowledge gathered from every quarter of the earth. From these causes they have become less automatic and servile. Independence of thought and of action is encouraged, originality favored and the stock of knowledge diffused, more critically analyzed and more practically applied. But these same causes have worked out for them a mediocrity from which they may not if they would escape. The general body has been leavened and is fermenting throughout its whole extent. The so-called country physicians and surgeons, as never before, are proclaiming their right to be heard. Their influence and authority are being steadily augmented from results approximating the up-to-date experience of the best physicians and surgeons in the largest medical centers where often their own original concepts and methods have been adopted as the most logical and useful. Provincialism in medicine has less detractors and perhaps arouses a somewhat jealous feeling in the pretentious and assertive purveyors of scientific knowledge in the metropolis.

Throughout the history of medicine, men of genius in the provinces have startled the profession with useful discoveries and inventions that have revolutionized its progress. These men were epoch-markers, not numerous comparatively. They were isolated examples of the divine afflatus that exceptionally distills its aroma in any circumstances. They were not representatives of provincial medicine which was serving far beneath them in the routine of a mediocrity that possessed few of the characteristics of that which now exists from daily interchange of ideas and intelligent co-operation. Sims, McDowell, Jenner, Harvey, Lawson Tait, Long and others by an irrepressible spontaneity of genius, stood erect above the common herd, in the face of social, legal and professional restraints and boldly delivered their message to the reigning authorities in the great cities from which the progressive teaching of the period was filtering slowly down into the general profession. Mediocrity was not so universal, scientific class distinction in medicine was broadly drawn, authority was dominant and severe, criticism less tolerated, custom more satisfying, consequently research and progress less ambitious and less indulged.

From time immemorial, authority in medicine had emanated from the cities and enslaved the mediocrity. Aristotle, the great naturalist, anatomist and evolutionist, had taught from Athens. Hippocrates, the judge-advocate, who finally divorced medicine from the superstitions of religion and philosophy, removed from Cos to Athens and Larissa, whence flowed the whelming current of his wisdom and influence. Celsus and Galen were supreme at Rome with a dogmatic control of the mediocrity that spanned the Middle Ages and held its sway long after the marvelous biological researches of Wolff, Pander, Baer, Schwann and Schleiden had been established as fundamental scientific facts not widely known and realized by a profession that might have been unshackled through their liberal investigation and acceptance. Even the discovery by Harvey that settled the fanciful and discordant ideas concerning the blood circulation and that of Aselli of the lymphatics, abruptly reforming the crude physiology and pathology of Galen, did not suffice radically to supplant his therapeutic views which subdued a pliant mediocrity for more than a century afterwards, indeed exerting a baneful influence to a much later period. The timely and salutary warnings of Sydenham, Chambers, Todd and Bennett seldom prevailed against the Galenic philosophy. Well known and appreciated by many, the mediocrity was inadequately impressed. *Contrarii, contraris, curanter* symbolized in medicine the besotted radicalism of the times; neither rest nor supplies should be given to a *materies morbi* that must be driven out of the stronghold by the most heroic measures taking little heed of strategical delay or of watchful conservatism. System replaced system with its dictatorial pronouncement "Eureka!" to be echoed by a submissive following that infrequently rebelled with success, or even acknowledgment. Each system was up-to-date and was grasped imperfectly yet loyally by a mediocrity straggling in the rear only to arrive at the front to find a new Utopia with its new commands—"The theory of today was the heresy of tomorrow." For long this was the up-to-dateness of the mediocrity and kept wide the distance between the pioneer reformers and the greater portion of a remote and uninstructed mass.

"The times are changed and we are changed with them." "There is nothing constant but change," but the change is an epigenesis built of universal, more deliberate research and its skilled utilization. An up-to-date, enlightened mediocrity is the Herculean factor in the wise-doing of the world's work; it has come into its own. It is the labor of Wolff, Jenner, Noeggerath and Semmelweiss quickly and fully recognized by its immediate proof and application to the needs of humanity. It is the Cargyle of Arkansas, McReynolds of Texas, Matas of Louisiana, the Mayos of Minnesota and others of "common clay" that might be mentioned. In truth, mediocrity never should have been thought degraded. Rightly consider the expert agriculturist and the laboring man in all ages of the world's progress! They are the basic pillars of our civilization; they have ever wrought the plasm that has initiated and enlarged the mentality of every man of letters, science and statesmanship. The colossal pyramids of Egypt securely rose upon the granaries of the fertile Nile valley. The splendor and wealth of Croesus was but the output of his incomparable agricultural domain. The great medical school of Alexandria, itself, was only a sequel of the avarice of Alexander tempted by a beauteous and productive land for his country's ever increasing mediocrity; and for the same reason, Rome forced her way into the Eastern and Western world setting an example, in modern times, to envious rulers and nations. The gorgeous hanging gardens and towering walls and palaces of Babylon emerged from the fruitful soil of the mythical Garden of Eden—a legacy of Summerian conquest and early development that gave birth, if not to Egyptian, certainly to our Jewish civilization. The gentle-minded Horace and the sentimental Virgil revelled in the marvelous works of Nature as unfolded by their handiwork, and Cicero stood appalled, but with delight, at his laborious training of the luxuriant yet mysterious vine.

Science may harness the electrical current for its uses and confine the vaporous elements of steam to expand the comforts and pleasures of life, but "It cannot make a buttercup," nor form in the brain of the man of science and of art the seed that grows the skill and power to conceive and execute. It is labor that preserves and develops the seed that supplies the intellectual plasm, nay! the life unit itself of man; but this labor, in ancient times, was mediocrity, *not* up-to-date. The expert labor of the modern artisan and agriculturist is paralleled by that of the modern physician and surgeon; it is mediocrity up-to-date in its wide dissemination and efficiency. It was up-to-date mediocrity, new-born and transmitted, that swept away the feudalism and clerical tyranny of the Middle Ages, and that later, in France and America, engaged in revolution to promote a growing sense of man's rightful claim to civic and religious freedom.

It may need not the fitful outburst of asserted genius to perfect its work; for various are the definitions and manifestations of this genius. The apple fell at the feet of Newton and from this fact evolved the immutable law of gravitation. Samuel Brown was obstructed in his morning's walk by a spider's web subarched across the path; the suspension bridge arose in all its grandeur and usefulness. In the Cathedral of Pisa a swinging lamp evoked the genius of Galileo; since when the pendulum has measured the seconds, the minutes and the hours of the day. The horny head of a fragile shipworm bores an archway into the wood; Brunel observed this and constructed the Thames tunnel on similar lines. In Gloucestershire the milkmaids that had taken cow-pox convinced Jenner that they were secure against the most dreaded disease; vaccination has eliminated almost the direful scourge from the sight of gods and men. Galvani's frogs' legs lay upon a metal plate and their twitching transmutes darkness into light and "puts a girdle round the globe." Sims' great opportunity lay in the handle of a spoon; the exalted emblem of our modern gynecology. The brutal application of a heated iron constricted the vessels but the merciful and more lasting constriction by Ambrose Pare is the simple ligature. Likewise, this is a quality of genius that animates and strengthens our now up-to-date mediocrity. Witchery, faith cures and phylacteries are explained and miracles are solecisms. "We surrender not our judgment to the fascination of a name;" not to the prison walls of an abject credulity nor superstition. Induction and theory must base upon a substantial warrant in the facts of anatomy, physiology, chemistry and pathology as revealed by astute and repeated observation; for herein abide our faith and our works. "The assertion which outstrips evidence is not only a blunder but a crime" and "Not for golden fancies iron truths make room." We know nothing of the "categorical imperative" in our scientific no less than in our moral development that is ordered by heredity, time and conditions; nor fear we "bell, book and candle" in our labors for and declaration of the truth. "Far from the madding crowd's ignoble strife" in wealth, politics and theology, we learn the variable status of our existence; its upbuilding and its destructive forces. "Our just perspective" discovers and relates all these, their due merit, harm and proportion as determined by our growth and surroundings. From maudlin sentiment and obsequious apathy we appeal to the irresistible divinity of reason and progress. We cannot mark time nor retreat, for behold a dubious struggle for life in the arena with brutal and hidden causes that make for woe, disorder and death whose grewsome light flashes the signal of despair; and not all the suppliants of age, station nor creed may stop its rhythm. But we "consult not the oracle of Trophonius," for science will know; we follow her tonight and kneel at the radiant shrine of Lucretius, of Aristotle and of William and John Hunter.

"Rally the scattered causes, and that line
Which nature twists be able to untwine."

This is the "open sesame" with its rich rewards, and this alone. It is this patient, plodding and independent research that is the benign result of our growing, up-to-date and competing mediocrity.

We question not that Minerva-like "freak of nature" that leaped forth a Homer, a Phidias, a Michael Angelo, a Beethoven, a Shakespeare and a George Eliot. But their intuitional faculties, if there be such, were a harmonious and potential blending of the imaginative and the observant; separated from the facts of nature even they could not have developed. Perhaps it was an ordinary obsession, disciplined by care and zeal, evoluting to the highest form that evolved such giants above the mediocrity. These anacronisms have appeared in Medicine and others shall come; we welcome their aid and inspiration; although we shall toil in the storehouse of Nature with unflagging energy and perseverance. Our strenuous legions will break over the arduous heights of science; for science, as was asserted of war by Napoleon, has no Alpine barriers that yield not the way. But we shall keep orderly the perennial fires of enthusiasm and like Palissy from his heated ovens, extract the polished wares of our life-saving work.

And our moral should be a reverence for that which has gone before—the sign-manual of our date and powers. What a wealth of knowledge and of force is thus conferred upon our mediocrity up-to-date! Shall we ignore or degrade our professional heredity with complacent assertion of up-to-date conceits in our medical philosophy? Has Medicine by its union with other sciences or differently, become a "biological sport" as Mendel claimed may follow curiously from intermarriage? If so, were not these preexistent factors so-called "has-beens" that make and sustain the living present? What were our fathers and what are our "reverend seigniors" now? Do they bequeath of error and of value as we also shall? But this value is the primordial germ of truth expanding among the uncertainties of life; it is the "germinal plasm" of the future. "Some call it Evolution and others call it God!" We are but this evolution from the past; probably the select and atavistic germ blossoms in us a more glorious fruition.

> "I sometimes think that never blows so red
> The Rose as where some buried Caesar bled;
> That every Hyacinth the Garden wears
> Dropt in its Lap from some once lovely Head;"

or wafted out upon the sea of life, it renews a greater potency in more congenial soil. Alexander nightly pillowed his head upon his sword and Homer's Iliad; and what, think you, absorbed the "little Corsican" after he entered the military school at Brienne? Was it Plutarch's Lives or a Pope Pius' version of the New Testament? "Milton did not disdain to build two of his finest poems on Burton's Anatomy of Melancholy," nor Shakespeare to help himself liberally out of the essays of Montaigne. Virchow must have reflected upon the researches of the neglected Wolff, and Lister admittedly found his lesson in the experiments of Pasteur and Tyndall, who, in turn, were antedated crudely by the old French farmer's wife that tended the infected grape in her homely vineyard. Does Osler herald the self-limitation of most diseases? Asclepiades, a friend of Cicero, "was the first to announce the doctrine of self-limitation of disease, asserting that the principal cure for a fever was the disease itself"—a startling modern doctrine. We boast the comparatively recent introduction into surgery of the plaster of Paris, yet a Greek prisoner in the army of Darius cured this monarch of a broken leg with exactly the same material. Ambrose Paré only rediscovered the ligature, for silk and animal ligatures were employed by surgeons in the time of Celsus; and what is the noxious humor of the blood doctrine but a prophetic hint of the pathology of the blood plasma recently outlined by Harry Campbell? The germ of the antiseptic method lay buried in the royal tombs of Egypt, but the idea reappears in the term itself as probably first employed by two old practitioners—Miner and Tuttle—in our revolutionary times; and *mirabile dictu!* "the contemporaries of Hippocrates were in the habit of dipping their instruments in boiling water." Reginald Fitz is the accredited pioneer in the operation for the removal of the appendix; may he not have played "goo-goo eyes" with the paper of Mellier who in 1826 accurately described the pathology of this organ with amazing reliance on his researches and their future development? Lawson Tait "bestrode the world like a colossus and we petty men walked under his huge legs and peeped about," but Simpson, Collins, Bernutz and Goupil supplied the pabulum that reared this mental prodigy of imperious voice and stature. It may be true that "the diagnosis of diseases of the great viscera by percussion was never known before Auerbrugger," but in the writings of Hippocrates, "The treatise on the seventh month foetus has been attributed to Diocles of Carystus," who "was the first to point to the distinction between pneumonia and pleurisy." Will you say that the academic and philosophic Auerbrugger was not familiar with this history, or that Diocles breathed not through the paper tube of Laennec, the inventor of the stethoscope? Our forefathers "builded better than they knew" and often "the rejected stone has become the head of the corner." Every well trained veteran in our mediocrity has a story to tell that yet may signalize an onward movement of the entire profession.

Let us join our reverence for many long forgotten worthies with a defence of our medical ethics which is enticed by the Greek-bearing gifts of the tradesman. Let us embrace the spiritual side of evolution; and if the "lowly Nazarene" wins not a full triumph in our lives, we may, as did the casuist Huxley, balance the rights of the Ego with a magnanimous altruism that should illumin the aggressive shield of this society with the inspiring ensign—The North Texas Medical Association, its scientific aims, its morally grounded ethics in a mediocrity up-to-date.

---

For Texas Medical Journal.

